# The quality of childbirth care in China: women’s voices: a qualitative study

**DOI:** 10.1186/s12884-015-0545-9

**Published:** 2015-05-14

**Authors:** Joanna Raven, Nynke van den Broek, Fangbiao Tao, Huang Kun, Rachel Tolhurst

**Affiliations:** 1 Department for International Public Health, Liverpool School of Tropical Medicine, Liverpool, UK; 2 Centre of Maternal and Newborn Health, Liverpool School of Tropical Medicine, Liverpool, UK; 3 School of Public Health, Anhui Medical University, Hefei, China

**Keywords:** China, Childbirth, Quality of care, User perspectives

## Abstract

**Background:**

In the context of improved utilisation of health care and outcomes, rapid socio-economic development and health system reform in China, it is timely to consider the quality of services. Data on quality of maternal health care as experienced by women is limited. This study explores women’s expectations and experiences of the quality of childbirth care in rural China.

**Methods:**

Thirty five semi-structured interviews and five focus group discussions were conducted with 69 women who had delivered in the past 12 months in hospitals in a rural County in Anhui Province. Data were transcribed, translated and analysed using the framework approach.

**Results:**

Hospital delivery was preferred because it was considered safe. Home delivery was uncommon and unsupported by the health system. Expectations such as having skilled providers and privacy during childbirth were met. However, most women reported lack of cleanliness, companionship during labour, pain relief, and opportunity to participate in decision making as poor aspects of care. Absence of pain relief is one reason why women may opt for a caesarean section.

**Conclusions:**

These findings illustrate that to improve quality of care it is crucial to build accountability and communication between providers, women and their families. Ensuring women’s participation in decision making needs to be addressed.

**Electronic supplementary material:**

The online version of this article (doi:10.1186/s12884-015-0545-9) contains supplementary material, which is available to authorized users.

## Background

Ensuring access to and availability of skilled birth attendance (SBA) and emergency obstetric care (EmOC) that is effective and of good quality are key strategies to help reduce maternal and new born mortality and morbidity [1, 2]. It is however important that increased coverage is matched with improved quality of care in order to influence health outcomes and to promote utilisation [3].

China has made great strides in reducing maternal and infant mortality. The national maternal mortality ratio has shown a steady decline from 141 per 100,000 live births in 1990 to 17 in 2013 [4] and infant mortality rate has fallen from 42 per 1,000 live births in 1990 to 11 in 2013 [5]. This success has been largely attributed to the strategy of increasing hospital delivery [6, 7]. However, urban–rural and regional differences remain; the poorest rural counties experience almost five times higher maternal mortality than urban areas [6]. Hospital delivery rates have risen steadily since 2004 throughout China, and in rural areas the proportion of women giving birth in a health facility rose from 69 % in 2003 to 96 % in 2011 [8]. In the context of improved health care availability and utilisation, rapid socio-economic development and health system reform, and increasing healthcare costs, it is imperative to now consider the quality of services [8].

In China, the maternal health care system is characterised by a three-tier service network [9]. At the county level, there are Maternal and Child Health hospitals, general hospitals and Traditional Chinese Medicine hospitals which provide antenatal, childbirth and postnatal services. At the township level, antenatal and postnatal care, and sometimes childbirth services are provided by Township hospitals. The township hospitals can be further divided into central hospitals that provide caesarean sections (CS), and basic hospitals which provide vaginal delivery services. At the village level, maternal health workers and doctors provide antenatal and postnatal care and can refer. The staff skill mix and division of labour for providing maternal health care services in rural areas is complex, with large variations between areas [10]. In the county-level hospitals, obstetricians provide antenatal and intrapartum care whilst midwives give antenatal care and assist with childbirth. Neither provides postnatal visits to women at home. Some township hospitals are staffed with obstetricians who provide antenatal, childbirth and postnatal care services, whereas others have midwives who provide these services.

There is no universally accepted definition or model of quality of care. It is widely acknowledged that quality is multi-faceted, incorporating a number of dimensions, including safety, effectiveness, patient-centeredness, and that a range of perspectives are relevant, including patients as well as health care providers and managers [11]. Approaches to quality of care that are specifically important for maternal and new born health include a rights based approach and the recognition that non-biomedical outcomes may be more important than for other areas of health care because childbirth is a culturally and emotionally sensitive area. The reproductive health rights approach advocated by Freedman [12], suggests looking at the applicability of rights in layers: the fulfilment of human rights means 24 h readiness in terms of availability of the necessary human resources, facilities, equipment and drugs, and the ability to mobilise these when needed; at the next layer, is how those services are delivered with a focus on human dignity and non-discrimination.

There is limited data available, particularly in low and middle income settings, on the quality of maternal health care. China, a rapidly expanding middle-income economy, is facing the challenge of over-medicalization of health care, including maternity care [8]; the pressure to generate operational costs and the fee-for service system appears to encourage a general overuse of technology and medicines, long lengths of inpatient stay and a high CS rate that is increasing in rural areas [8, 13, 14]. As such, understanding quality of care in this context may offer important lessons for developing maternity services in other transitional economies. In China, the focus of studies has largely been on availability of, access to and utilisation of services although some have implied a poor quality of maternal health care [9]. Two earlier studies have looked at some aspects of quality from the perspectives of policy makers, health care providers and women [15, 16]. Women’s expectations and experiences of childbirth services are an important part of the assessment of quality of care; no previous study specifically investigating these in China was identified. This study explores women’s perceptions and experiences of the quality of care during childbirth in a rural setting in China.

## Methods

As this was an in-depth exploration of the quality of childbirth care as experienced and understood by the women themselves, a qualitative research methodology was used. Semi- structured interviews and focus group discussions were employed.

This study was conducted in one rural county in Anhui Province. The county shows good performance in utilisation of childbirth services and maternal and infant outcomes, with figures slightly better than the national average for rural areas (Table 1). Six out of 18 townships in one county were chosen using the following criteria: distance from the county city (two townships were less than 20 km, two townships were between 20 and 40 km, and two townships were more than 40 km from the county city); level of township hospital (three townships had central hospitals and three townships had basic hospitals); and socio economic status (two townships with low, two townships with medium and two townships with high socio economic status). In addition, two county level hospitals were selected: the county hospital as it is the referral centre for the county, and the Traditional Chinese Medicine hospital as it is a popular choice amongst women for childbirth. The study took place between 2007 and 2009.

**Table 1:** Maternal and infant health and health care indicators for the study county and national rural areas in 2006

Indicators	Study county	National
Maternal mortality ratio (per 100000)	41^a^ (Anhui province)	45^d^
Infant mortality rate (per 1000)	13^b^	19^d^
% of facility based deliveries	99 %^c^	88 %^d^
% of Caesarean Section	46 %^c^	46 %^e^

^a^MMR in study county is not available; Anhui province data: United Nations Economic and Social Commission for Asia and the Pacific, Health Bureau of Anhui Province China: Multi-sectoral determinants of maternal mortality in Anhui Province China*.* UNESCAP. Accessed on June 16, 2011. Available: http://www.unescap.org/esid/psis/meetings/MMR/China.pdf

^b^County Health Bureau. County Health Statistics*.* Anhui, China: Ministry of Health County Health Bureau; 2007

^c^Huang K, Tao F, Faragher B, Raven J, Tolhurst R, Tang S, Broek Nvd: A mixed-method study of factors associated with differences in caesarean section rates at community level: The case of rural China. Midwifery 2013, 29(8):911–920

^d^Ministry of Health, China. Chinese Statistical Health Yearbook 2009. Ministry of Health China. Beijing: Ministry of Health, 2009. Accessed June 1, 2011. Available: http://tongji.cnki.net/overseas/Dig/Dig.aspx#

^e^Lumbiganon P, Laopaiboon M, Gülmezoglu AM, Souza JP, Taneepanichskul S, Ruyan P, Attygalle DE, Shrestha N, Mori R, Nguyen DH et al.: Method of delivery and pregnancy outcomes in Asia: the WHO global survey on maternal and perinatal health 2007–08. Lancet 2010, 375(9713):490–499

Thirty five semi-structured interviews and five focus group discussions (FGDs) were conducted with women who gave birth in the past 12 months in the selected eight facilities (six township and two county level hospitals). We selected some women for interviews to gain an in depth exploration of their experiences. Others were recruited to FGDs in order to understand the range of perceptions of quality as a concept and its application in available services. Women with a range of ages, parities, education levels, types and places of childbirth were purposively selected. The characteristics of women are described in Table 2. There were no women under the age of 20 years and more women were primiparous, reflecting the Family Planning Policy that is implemented in the study county, which encourages later marriage and childbearing, and restricts family size. Residency status refers to whether women stay in their registered home county to work (non-migrant) or migrate to other provinces or cities to work usually in factories or restaurants (migrant).

**Table 2:** Characteristics of women (n = 69)

Characteristic		Number
Age	≤20 years	0
	21-29 years	47
	≥30 year	21
	Unknown	1
Parity	Primiparous	45
	Multiparous	24
Residency status	Non migrant	36
	Migrant	33
Type of delivery	Normal Vaginal Delivery (NVD)	49
	Caesarean Section (CS)	20
Place of delivery	Township hospital	41
	County level hospital	28
Type and place of delivery	NVD in township hospital	32
	NVD in county hospital	17
	CS in township hospital	9
	CS in county hospital	11

Using topic guides, the interviewer or facilitator explored what quality in childbirth care means to women, their expectations and experiences of childbirth care. The health systems model of quality of care was used to develop the topic guides: perceptions of the quality of the structure of health care services, as well as the quality of the actual health care activities, and quality of the outcome were explored [17]. See Additional file 1 for the topic guides.

All interviews and discussions were conducted in Mandarin Chinese by the trained research team, tape recorded with consent, transcribed by the research team into Mandarin Chinese and then translated and checked for accuracy. All participants were given a small gift (washing powder) to compensate for their time.

Data were analysed using a framework approach which facilitates rigorous and transparent analysis [18]. The transcripts were read to identify emerging themes; a coding framework was developed based on these themes and all transcripts were coded with this framework; charts were created for all themes; these charts were used to describe similar and divergent perceptions, develop explanations and find associations between them. The computer programme “MAXqda” was used to support the analysis [19]. Thematic differences between the accounts of women delivering in the different types of facility were explored. However, there were no strong thematic differences between the accounts of women delivering in the two types of county level facility. Therefore we have focused in our analysis on the differences between county and township levels which emerged strongly.

Ethical approval and permission for the study were gained from the Research Ethics Committees at the Liverpool School of Tropical Medicine in the UK (Approval no. 06.42) and Anhui Medical University (Approval No. 2007002). Permission was also obtained from the local county health bureau. Informed consent was obtained from each woman prior to starting the interviews and discussions.

## Results

The findings are presented as a comparison between women’s perceptions of what constitutes quality of care and their reflections on their actual experiences of childbirth across the following themes: place of childbirth; type and experience of childbirth; skills of birth attendants; communication between women and birth attendants; and role of family. Table 3 provides a summary of women’s perceptions and experiences.

**Table 3:** Summary of women’s perceptions and experiences of childbirth

Domains	Perceptions of quality	Actual experiences
Place of childbirth	In hospital.	Majority delivered in township hospitals because:
	County or higher level hospital because:	• Close to home
	• Comfortable, clean and quiet environment	• Doctors are good at NVDs
	• Good equipment	• Knew doctors because of previous delivery or antenatal care
	• Skilled providers who can manage emergencies	Environment:
	Environment:	• Township hospitals: rooms, beds and linen were dirty
	• Quiet and clean	• County hospitals: Labour and delivery rooms and wards were clean
		• Both: noise from streets and corridors; shared room and toilets
Type of childbirth	NVD:	NVD:
	• Quicker recovery	• Stay in hospital is shorter than for CS
	• Less pain following NVD	• Fewer complications and quicker recovery
	• Improves immunity and intelligence of baby	• First baby was NVD
		• Less pain with NVD
Experience of childbirth	• Pain relief which is safe for mother and baby	• Almost all women having NVD did not receive pain relief
		• Ill prepared for management of pain
		• Received oxytocin infusions to speed up labour and relieve pain
Skills of birth attendants	• Cause little pain; able to comfort and relieve pain	Majority viewed skills as being good:
	• Good at watching progress	• Careful, gentle and quick at doing examinations, deliveries and suturing
	• Disinfection of equipment and materials	
	• Care for mother and baby after delivery	• Putting up IV fluids
	• Older, experienced and confident	
Communication between women and birth attendants	• Treat with respect:	• Township hospitals: most received support from doctors
	◦ Showing concern	• County hospitals: most received little/no support from staff
	◦ Being kind and friendly	• Both: Doctors did not explain what care they were providing; gave no choice about care; and did not ask consent for investigations or interventions
	◦ Giving information about care	
	◦ Seeking women’s views and consent	
Role of family	Husband and other relatives present for childbirth to provide support and encouragement and deal with problems	Township hospitals: all women had relatives present for NVD
		County hospitals: only half of women had relatives present

### Place of childbirth

The majority of women identified the importance of safety of care resulting in a healthy mother and baby, as the principal aspect of quality of care. Almost all women perceived hospitals to be safer places for childbirth than home as health care providers are immediately available to deal with any problems. They also reported that home deliveries were no longer permitted by local authorities. However, a few women wanted to give birth at home because this would afford greater privacy.

*“Quality of care means safety of mothers and babies. There must be enough equipment in the delivery room, there should be more doctors on at night in case of emergency.” Woman with NVD, township hospital.*

Most women expressed a preference for county level hospitals as these were perceived to be comfortable, clean, quiet, and well equipped, with skilled health care providers able to manage emergencies. However, in practice, most women gave birth in the township hospitals because: they were closer to home; the doctors were seen as good at conducting a normal vaginal delivery (NVD) (as opposed to only a CS); they had received antenatal care or previous childbirth care there; and speed of onset of labour prevented them from travelling to the county hospital.

*“My mother in law said that the doctors here know a lot about how to look after women and their babies. During pregnancy this doctor always examined me. I won’t worry if I give birth here. I have not been to the county hospital, but I heard that the attitudes of the staff are not good.” Woman with NVD, township hospital.*

*“My mother in law said that we have had a baby already, and I was over 30 years old, in case of an emergency, we chose to go to the county as it has better equipment and skilled doctor.” Woman with NVD, county hospital.*

Many women were unhappy with the cleanliness of the rooms and bed linen in township hospitals, whilst those who gave birth in county hospitals generally perceived the rooms as clean. In both settings delivery rooms and wards were reported to be noisy and there were no adjacent toilets or bathrooms. However, most women were satisfied with the level of privacy provided. Only hospital staff or relatives were allowed into the labour and delivery rooms, and doors, windows and curtains were kept closed.

*“It is not clean. It should be. There were many mosquitoes. The hospital should know that infection can be caused by sanitation. The county hospital is better and other big hospitals are cleaner.” Woman with NVD, township hospital.*

### Type and experience of childbirth

The majority of women perceived a NVD as the ideal. Their reasons for this were: quicker recovery and shorter stay in hospital; less pain following childbirth; better outcomes for the baby as it improves immunity and intelligence; and previous childbirths were normal. Only a minority reported CS as the ideal mode of childbirth. They perceived a CS as less painful as anaesthesia is given; they fear the pain of labour and childbirth; they can have tubal ligation at the same time; and they can choose an ‘auspicious’ date of birth.

*“I want to give birth by myself at first, but I’m afraid of pain, and chose to have CS delivery.” Woman with CS, county hospital.*

The decision making process regarding choice of type of childbirth appears to be complex. Some women reported making the decision themselves based on their own knowledge and information seeking. For example, one woman requested a CS as she had read that large babies need to be delivered by a CS and the ultrasound scan showed her baby was large. Other women were advised by their doctors to have CS for medical reasons. However, some women said that although doctors suggested having CS because of complications with the baby such as cord around the baby’s neck, they felt that the doctors insisted in carrying out the operation as they wanted to earn more money. Other women followed the advice of family members or friends.

*“I got information from a book to say normal delivery is good for uterus contraction. I chose a normal delivery. I was afraid about the operation. The doctor examined me and suggested to have CS, but I wanted to deliver normally.” Woman with NVD, township hospital.*

*“I did not discuss it with the doctor. I knew that I needed a caesarean section because I had read things and listened to other women talking. I have the telephone number of a doctor who does CS.” Woman with CS, county hospital.*

All but one of the women who had a vaginal delivery were not offered any form of pain relief (in one case the woman was offered paracetamol). Most women thought that labour pain was ‘natural’ and could not be relieved. Despite this, they wanted help to relieve the pain but thought that drugs could harm the baby. Some women thought that an oxytocin infusion could help to relieve pain by reducing the labour time. In practice, women generally felt ill prepared for management of pain; this was not discussed during antenatal care or education classes which occur in most facilities. Women’s main source of information was the internet and magazines. Many women did have oxytocin infusions. Some women reported requesting a CS or oxytocin infusion because of severe pain but said the doctors refused this.

*“I hope that when I feel pain I can have some pain relief, but this is not possible. Otherwise there would not be so many people choosing caesarean section.” Woman with CS, county hospital.*

*“I wanted pain relief but I think there were no medications and other methods to relieve pain in this hospital. I don’t know if it’s the same in other hospitals.” Woman with NVD, township hospital.*

### Skills of birth attendants

Most women reported that it was important that the attendant was skilled, including being: gentle during suturing and examinations; causing little pain and no injury; watching the progress of labour; ‘listening to’ the foetal heart; disinfecting equipment and materials; giving intravenous fluids efficiently; and managing both mother and baby well after delivery. Most women thought that the ideal birth attendant should be female, and older as they were seen as more experienced and skilled, and able to manage emergencies.

*“They should be good at watching labour, doing the delivery, and abdominal delivery. Very good skills. The doctor must not hurry and can control everything, making me feel relaxed.” Women with NVD, township hospital.*

Many women reported that in their experience the doctors and nurses were skilled. A few women who gave birth in the county facilities reported examples of poor care: allowing the perineum to tear badly causing great pain; not dealing with the umbilical cord after delivery; and not attending to the woman after childbirth.

*“She worked very quickly and she was experienced. After childbirth, she also asked the situation of me and my baby. I had a lot of bleeding after delivery and she saw me several times in case of postnatal haemorrhage. She also advised on caring for the baby.” Woman with NVD, township hospital.*

*“I felt all skills were good. For example, it was good when they were suturing me. Their actions were light and soft.” Woman with NVD, township hospital.*

### Communication between women and birth attendants

Most women preferred attendants who were confident, could make them feel relaxed, comfort them during labour, and “be responsible” (show concern and commitment to providing care) for women and their babies. Treating women with respect was a particularly important attribute, demonstrated through: showing concern; being kind and friendly; giving information; seeking women’s views; and treating everyone equally.

*“I hope the doctor could explain clearly to us, in order to calm us, and then we prepare for the delivery better.” Woman with NVD, county hospital.*

Most women reported that doctors in both township and county hospitals asked women and their families what type of childbirth they wanted and followed their wishes. However, doctors did not explain and women were not given a choice about other aspects of care they received, such as investigations, internal examinations, episiotomies or shaving of the perineum. As a result, they did not clearly understand what was happening.

*“Doctors should seek the views of mothers for most things. But they normally do what they want to. I haven’t the right to choose what care I receive.” Woman with NVD, township hospital.*

Most women who gave birth in the township hospitals, received support from the doctors, who helped them manage the pain, through encouragement, distraction, teaching them how to breathe deeply and massaging the abdomen and back.

*“The doctor said not to hurry, it must hurt when you deliver. It was painful, and she chatted with me. She always comforted me. It’s enough.” Woman with NVD, township hospital.*

However, most women giving birth in the county hospitals reported receiving little support from doctors or nurses. Staff did not comfort them, but told them not to cry out in pain as this upset other women. Doctors wanted women to give birth quickly, and blamed them for slow progress of labour; one woman reported being slapped. The nurses were unfriendly, and did not care for women.

*“Doctors pay less attention to you in the county hospitals. Doctors will not always stay with us, she may do some other things and observe us occasionally. She just does the delivery after I lie on the delivery bed.” Woman with NVD, county hospital.*

### Role of family

The majority of women wished that their husband, mother or other relatives be present during labour and childbirth as they could provide support and security and help them to relax. In practice, most women reported being allowed relatives to accompany them in the township hospital delivery rooms. Relatives gave encouragement and support which enabled women to deal with their fear and helped manage pain. Only half of women who gave birth in the county hospitals were allowed to have female relatives present during childbirth.

*“My mother and my husband were there from the labour until I gave birth to the baby. I could bear the pain when my husband and mother were with me, otherwise I cannot.” Woman with NVD, township hospital.*

## Discussion

This study has examined women’s expectations and experiences of childbirth care in rural China. This is the first study that has documented women’s assessment of quality of childbirth care in a rural setting in China: a sensitive topic in any setting, but even more so in the highly controlled environment of rural China. Quality of care was seen mainly in terms of “safety” which includes the availability of skilled medical personnel. Women’s expectations were met in terms of giving birth in hospital and level of privacy provided during care. However, other aspects of quality were not met, including giving birth in the hospital of choice, lack of management of labour pain, relatives not allowed to provide support during labour, and health care providers neither providing information to women nor involving them in decision making about care.

Women identified the importance of safety of care resulting in a healthy mother and baby, as the principal aspect of quality of care. This study supports the characterization of the dominant approach to childbirth in China is medicalization, and this affects women’s expectations of care. This medicalization is apparent in the preference for place of childbirth: despite most women expressing their preference for place of childbirth being at county and higher level hospitals, they gave birth in township level facilities. There are tensions between wanting technically advanced (medicalized) facilities and wanting a normal vaginal delivery, knowing the health care providers and being assured of companionship during labour. Other studies have shown that women bypass lower level facilities to deliver in hospitals where they perceive a better technical quality of care to be provided, but poorer interpersonal quality [20–22].

Although most women reported wanting pain relief, none received any pain relief drugs, in line with other studies in China [15, 23]. Although each woman’s experience of childbirth is unique, how women perceive pain and their coping mechanisms to manage pain is culturally defined [24, 25]. There are culturally proscribed behaviours towards pain: labour pain as an expected and normal part of giving birth was described in this study, as in other studies in Taiwan and Eastern China [25, 26].

Women’s ability to manage labour pain is influenced by how prepared they are for childbirth. In this study, women were generally ill prepared, with little explanation from health care providers about what to expect during labour and delivery. A systematic review of the effects of individual or group antenatal education for childbirth on pain among other outcomes did not provide sufficient evidence to support or oppose education [27]. However qualitative studies identified benefits including reducing anxiety and learning skills to help manage pain [28–30].

Women, particularly in the county level facilities, received little support from relatives or health care providers. Yet, personal and professional support during labour is critical. Women’s responses to childbirth pain may be modified by support received from caregivers and companions [31, 32]. Continuous support during childbirth reduces the need for pain relief and medical interventions [32]. How labour pain is managed appears to have far-reaching implications. Although the reasons for high CS rate are many and complex in China, such as the increasing age of primiparous women, the one child policy, women's expectations and understanding of safety, the financial benefits of opting for CS, professional interests and skills [13], labour pain and its management are important influencing factors identified by women in this study. During pregnancy, women reported fear of labour pain and decided to have a CS, which was seen as painless. Other women reported choosing CS during labour as they could not manage the pain without preparation or support and they feared for their own and the baby’s safety. Studies in China and elsewhere have indicated that fear of labour pain is an important influencing factor on choice of mode of childbirth [23, 33–36]. Several issues about the rising CS have emerged from the Chinese setting. Firstly, there are increased costs associated with CS for not only families, but the health care system too [8]. This is a particular problem for low income households where CS can account for 1/3 of the annual income [37]. Secondly, an excessively high CS rate can both result from poor quality of care and also further undermine that quality from both clinical and interpersonal perspectives. From the clinical perspective, limited skills and confidence in monitoring labour and identifying when intervention is needed, can increase CS rates [13]. There is also a risk that confidence to manage NVDs diminishes when these skills are used less frequently due to high CS rates. This is supported by findings from a study of emergency obstetric care in Shanxi province in China showing providers’ loss of confidence in dealing with complications [38]. From the interpersonal perspective, satisfaction with childbirth is not reliant on the absence of pain. Women may view pain as a necessary part of the birth experience and may be evaluated positively when women feel a sense of achievement [31, 39]. Participation in decision making and the amount and quality of support from providers are important dimensions of satisfaction with childbirth and may override other influences including pain and childbirth preparation [39]. Providing caring support and respect for women’s dignity and autonomy is therefore a central aspect of good quality care and may also reduce demand for CS in this context. Exploring providers’ perspectives of management of labour pain, as well as operational research on antenatal preparation and supportive management of labour are needed to develop guidelines and inform training.

This study found that women had little opportunity to participate in decision making about their care. This may not be surprising, given the complicated issue of individual informed consent in China. The western concept of respect for the person as an autonomous individual supporting the argument for informed consent may not be applied to Chinese society in exactly the same way [40]. Social roles in China are chiefly defined by familial and institutional relations where people exist through and are defined by their hierarchical relationships with others. Medical decisions may not be considered simply from an individual’s perspective but as embedded in a web of social relationships with far-reaching effects [40]. There is also an underlying obedience to authority, with doctors and officials having high status within society [41]. There is a need to understand the complexity of decision making and informed consent in the sensitive area of maternal health from the perspective of women, families and health professionals.

There are several limitations to the study. Firstly, observation as a method to gain additional insights into the culture of quality in the study sites, including interactions between providers and women was planned, but providers were not willing to participate. Secondly, this was a retrospective study relying on women’s recall of events. Exploring pregnant women’s expectations of quality of care and interviewing them in the postnatal period for their experiences and perceptions of care would generate a more detailed picture of quality of care.

## Conclusions

Listening to women’s voices about their expectations and experiences of care is vital if we are truly committed to providing good quality care. This study illustrates the need to build accountability and communication between providers and women and their families for improved quality of care including informed consent and decision making. This has implications for continued clinical quality, costs to women and their families as well as to the health system, and for birth outcomes in the widest sense.

## Additional file

Additional file 1:
**Topic guides.**
