# Supplementary material for: The quality of childbirth care in China: women’s voices: a qualitative study
Source: BMC Pregnancy Childbirth. 2015 May 14;15:113. doi: 10.1186/s12884-015-0545-9 (PMC4457993; doi:10.1186/s12884-015-0545-9)
Supplement: Additional file 1: — Topic guides. [file 12884_2015_545_MOESM1_ESM.doc]

**Annex 1: Topic guides**

**Focus group discussion with women**

1. What does quality of care mean to you when we talk about childbirth?
2. What do you think an ideal childbirth would be like?

Areas for probing:

- Type of delivery
- Place of delivery
- Environment
- Support

1. What would you like the person doing the delivery to be like?

Areas for probing:

- Gender
- Skills / qualifications
- Attitudes

1. Tell us about your experiences of care during childbirth (during labour and delivery)
2. What would you say is good about the care you received?

Areas for probing

- Communication / support
- Skills of staff
- Environment
- Procedures during childbirth

1. What areas of care would you like to be improved?

Areas for probing

- Communication / support
- Skills of staff
- Environment
- Procedures during childbirth

1. Is there anything else you would like to add?

S**emi structured interview with women**

1. Ask the woman to talk briefly about her baby.
2. How do you prepare for childbirth?

- Where do you get any information about childbirth? (reading, internet, health workers, friends, family). What do you talk about?
- How do you prepare financially for delivery? What do you prepare for? How did you decide what to save money for?
- How do you and your family organise transport, looking after the family and home, work duties?

1. How do you prepare for emergency situations?

- Do you discuss with your family what you would do in an emergency during pregnancy (e.g. pre-term delivery or bleeding)? What do you discuss?
- Do you discuss with your family what you would do in an emergency during delivery? What do you discuss?

1. Before you gave birth, what did you think an ideal childbirth would be like?

- Ideally, where would you like to give birth?
- Ideally, who would you like to be present?
- Ideally, how would you like to give birth?

1. What would you like the person doing the delivery to be like?
   - What skills would you like them to have?
   - What qualifications would you like them to have?
2. Tell us about your experiences of care during childbirth from when you arrive at the hospital until after you have given birth

Areas for probing:

- Administration and investigations when enter hospital
- Communication
- Skills of the staff
- Privacy
- Environment and equipment
- Experiencing procedures during delivery
- Support from staff
- Support from family
- Mobilisation and position for delivery
- Pain management

1. What would you say is good about the care you received?
2. What areas of care would you like to be improved?
3. We have talked a lot about your experiences of childbirth, so to conclude what does quality of care mean to you when we talk about childbirth?
4. Is there anything else you would like to add?
